# Malignant Transformation of Retroauricular Hidradenoma in Hidradenocarcinoma in a Nine-Year-Old Patient: A Case Report

**DOI:** 10.7759/cureus.37160

**Published:** 2023-04-05

**Authors:** Sara Zorro, Rafael Matias, Cátia Sousa, Artur Aguiar

**Affiliations:** 1 Radiation Oncology, Instituto Português de Oncologia do Porto, Porto, PRT; 2 Pediatric Oncology, Instituto Português de Oncologia do Porto, Porto, PRT

**Keywords:** pediatric oncology, hidradenoma, vmat, radiation therapy, hidradenocarcinoma

## Abstract

Hidradenocarcinoma, a rare malignant entity that derives from sweat glands, is especially rare in the pediatric population. The treatment of choice is surgery. Radiation therapy is used only in selected patients. Chemotherapy is not used extensively because its effectiveness has not been demonstrated yet.

This case report describes a nine-year-old female patient who presented in 2018 with a vegetative lesion in the right parietal region. After excisional surgery, pathology confirmed that the lesion was a benign hidradenoma. However, the lesion recurred six months later, and subsequent surgery revealed nodular hidradenoma with positive margins. In July 2019, a new heterogenous lesion appeared in the right retroauricular region, which was surgically removed. The pathology report found possible malignant characteristics, and the patient was referred to our hospital where she was diagnosed with poorly differentiated right retroauricular carcinoma with infiltrative and perineural permeation, along with homolateral lymph node metastasis. It was histologically compatible with a hidradenocarcinoma. The patient underwent a wide-margin excision and homolateral total cervical lymphadenectomy, followed by adjuvant radiotherapy. The last follow-up MRI was negative for disease recurrence or metastasis; however, a slow-growing node on the left jugular chain (level II) was noted. The patient is on regular follow-ups to monitor disease status and treatment-related adverse events.

This case highlights the challenges of diagnosing and treating hidradenocarcinoma, a rare malignancy that requires aggressive management with a multidisciplinary approach. More robust clinical evidence is needed to define the best treatment approach for these aggressive tumors.

## Introduction

Hidradenocarcinoma (HC) consists of a rare malignant entity that derives from sweat glands, accounting for only about 6% of all malignant eccrine tumors [1]. Incidence is higher in patients 60 years or older and very rare in pediatric patients. The most common location described is the head and neck, followed by the limbs. Some studies have reported no significant difference in incidence according to sex while others have [2]. Although most of these tumors arise de novo, some can also develop from a pre-existing hidradenoma [1,3]. According to histological classification, the recognized variants include nodular hidradenocarcinoma, malignant acrospiroma, malignant clear-cell hidradenoma, and clear-cell eccrine carcinoma [1]. Local and frequent recurrences are common in the natural course of these tumors [4]. The involvement of regional lymph nodes and the presence of distant metastases are associated with a poor prognosis. In the case of metastatic disease, the five-year survival drops to less than 50% [5].

The treatment of choice for HC is surgery consisting of wide local excision with negative margins, with or without lymph node dissection. Radiation therapy (RT) is used in selected patients. Chemotherapy (ChT) is not used extensively because its effectiveness has not been demonstrated yet [1,4,5]. Local recurrence rates following surgery range from 10% to 50%. Radiotherapy is usually not proposed as a first-intention therapy, but radiation therapy is necessary when surgery is impossible or in case of incomplete primary surgery. High doses ranging from 50 to 70 Gy are recommended [1,6,7].

Due to its rarity, original data published consisting of only a few patients possibly led to misleading and unreal conclusions about the characteristics of this entity. A review of a higher number of patients would allow a better understanding of the clinicopathological characteristics of HC.

## Case presentation

This case reports a nine-year-old female patient followed in the outpatient department of a major pediatric hospital since March 2018 due to a vegetative lesion in the right parietal region, originally 8 × 6 mm in dimensions. A few weeks after admission, excisional surgery was performed. Pathology concluded a benign tumor with characteristics of hidradenoma, without signs of malignancy, with a depth of 9 mm and positive lateral and deep margins.

Six months later, the lesion recurred, and due to its original benign nature, the patient was kept on vigilance until February 2019. The lesion continued to grow and was again surgically removed. Pathology was consistent for nodular hidradenoma, with lateral and deep margins inferior to 1 mm.

In July of the same year, there was a new palpable heterogenous lesion on the right retroauricular region, measuring 40 × 30 mm, and an ipsilateral swollen lymph node. Again, the fast growth justified a new surgical approach. This time, the pathology report found possible malignant characteristics with aggressive behavior, such as low differentiation, infiltrative characteristics, and perineural permeation. The surgery was considered incomplete with positive margins.

The patient was referred to our hospital in late 2019 with the diagnosis of poorly differentiated right retroauricular carcinoma, with infiltrative and perineural permeation, and immunohistochemically positive for cytokeratin MNF116, p63 and p40, and negative for S100, cytokeratin 7, and NUT. Kreyberg and Alcian blue failed to demonstrate the presence of intracytoplasmic mucin or goblet cells. The histological features were compatible with hidradenocarcinoma with areas of squamous, trichilemmal, and ductal differentiation. There were two palpable swollen lymph nodes, measuring 1.5 cm each, in the right and left cervical areas.

**Figure 1:**
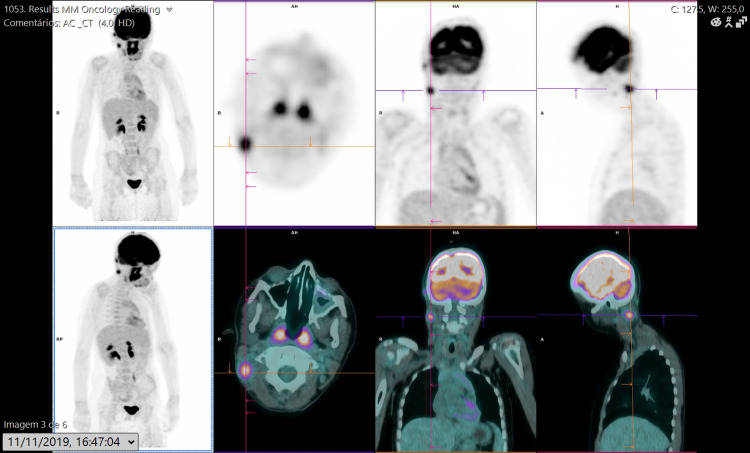
Positron emission tomography scan in November 2019.

The staging positron emission tomography (PET) scan (Figure 1) and computed tomography (CT) scan showed multiple cervical adenopathies distributed along the Va level (maximum standardized uptake value: 6.95), submandibular region, and the jugular chain.

**Figure 2:**
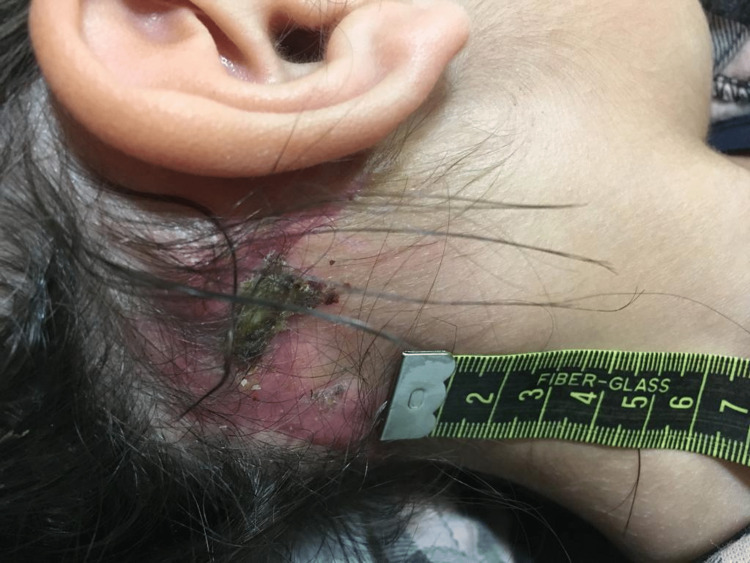
Retroauricular lesion before resection and reconstruction in November 2019.

She was submitted to a wide-margin excision along with homolateral total cervical lymphadenectomy (43 excised lymph nodes, from levels I, IIb, III, IV, and V), followed by adjuvant RT. The resection occurred in November 2019 (Figure 2) and was followed by reconstruction using a temporoparietal galeal flap and skin graft from the thigh. The histological examination reported an undifferentiated carcinoma with extension into skeletal muscle tissue. There was no evidence of lymphovascular or perineural permeation and no involvement of locoregional lymph nodes or resection margins.

**Figure 3:**
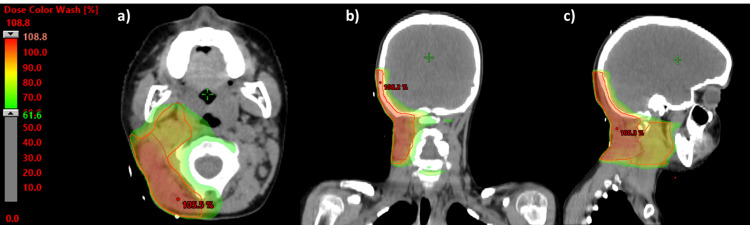
The orange line within the dosimetric plan corresponds to PTV 54 Gy and the blue line corresponds to PTV 60 Gy. The color wash threshold was set to 61.6% of the total dose. Isodose curves of the VMAT treatment plan. (a) Axial plane; (b) coronal plane; (c) sagittal plane. PTV: planning target volume; VMAT: volumetric-modulated arc therapy

For RT, a CT simulation for treatment planning was performed, and the images were fused with diagnostic images of CT and PET. Retroauricular tumor bed (scar plus pre-surgery tumor volume) and right level II nodes were defined as the clinical target volume (CTV) on simulation CT. The planning tumor volume (PTV) was defined as 0.5 cm margin expansion from the CTV, and a total dose of 60 Gy in 30 fractions was prescribed (54 Gy, 1.8 Gy/day to the tumor bed and right level II nodes plus the tumor bed boost of 60 Gy, 2 Gy/day). A volumetric-modulated arc therapy (VMAT) with a simultaneously integrated boost (SIB) technique was used (Figure 3).

**Figure 4:**
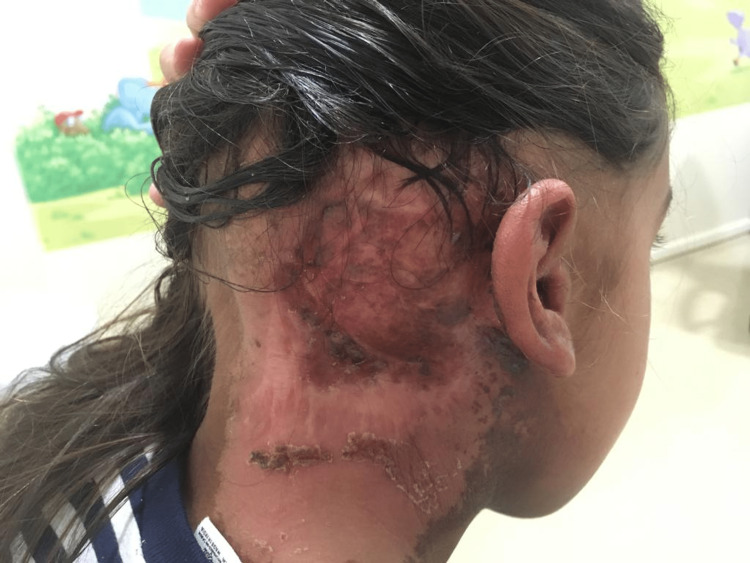
Retroauricular lesion after radiation therapy in March 2020.

The patient was treated for 40 days, between January and February 2020, with no interruptions (Figure 4). During treatment sessions, she experienced mucositis, odynophagia (Radiation Therapy Oncology Group (RTOG) - grade 1), and dermatitis (RTOG - grade 2) that resolved with support medication.

**Figure 5:**
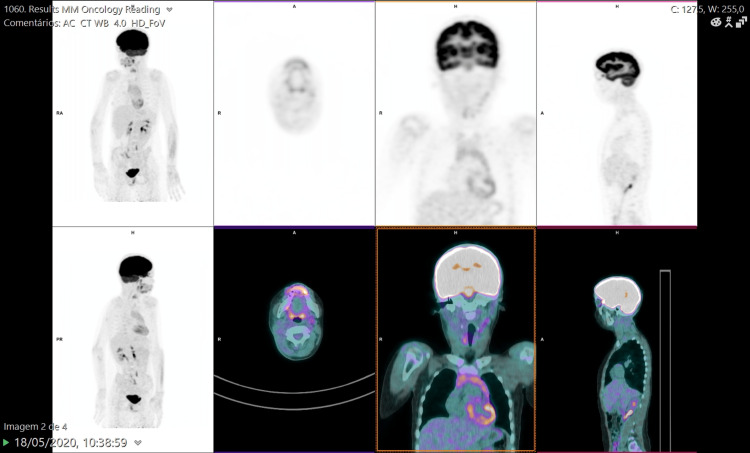
Positron emission tomography scan in May 2020.

In May of 2020, the follow-up PET scan was negative for disease recurrence or metastasis (Figure 5).

**Figure 6:**
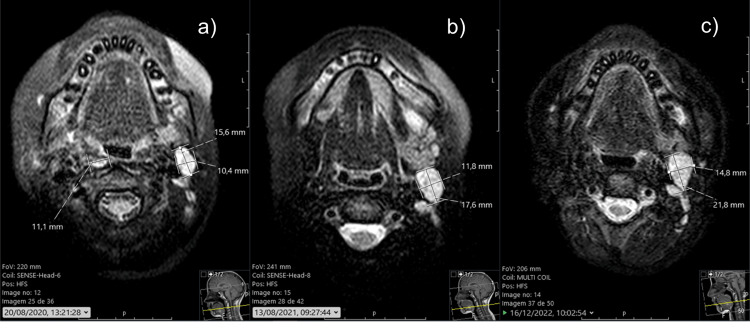
(a) MRI in August 2020; (b) MRI in August 2021; and (c) MRI in December 2022.

Three months later, in August 2020, a follow-up MRI (Figure 6a) was negative for disease recurrence or metastasis but showed multiple nodes along the left jugular chain (level II), with the largest measuring 16 × 10 mm. In August 2021, a follow-up magnetic resonance imaging (MRI) (Figure 6b) showed the same multiple nodes along the left jugular chain (level II), with the largest now measuring 18 × 12 mm; three retropharyngeal lymph node conglomerates, with the largest measuring 9 mm; and one node in the right submandibular region measuring 8 mm. Three months later, the patient was submitted to an ultrasound-guided needle biopsy of a left level II node. The specimen was negative for metastasis, and she remained in vigilance. In December 2022, the MRI (Figure 6c) was negative for disease recurrence or metastasis; however, one of the left jugular chain (level II) nodes was now approximately 22 × 15 mm in size.

Considering the negative lymph node biopsy and to maintain close monitoring of the cervical nodes, an ultrasound was performed in January 2023, which reported the stability of the lymph nodes previously identified currently measuring 22 × 9 mm.

**Figure 7:**
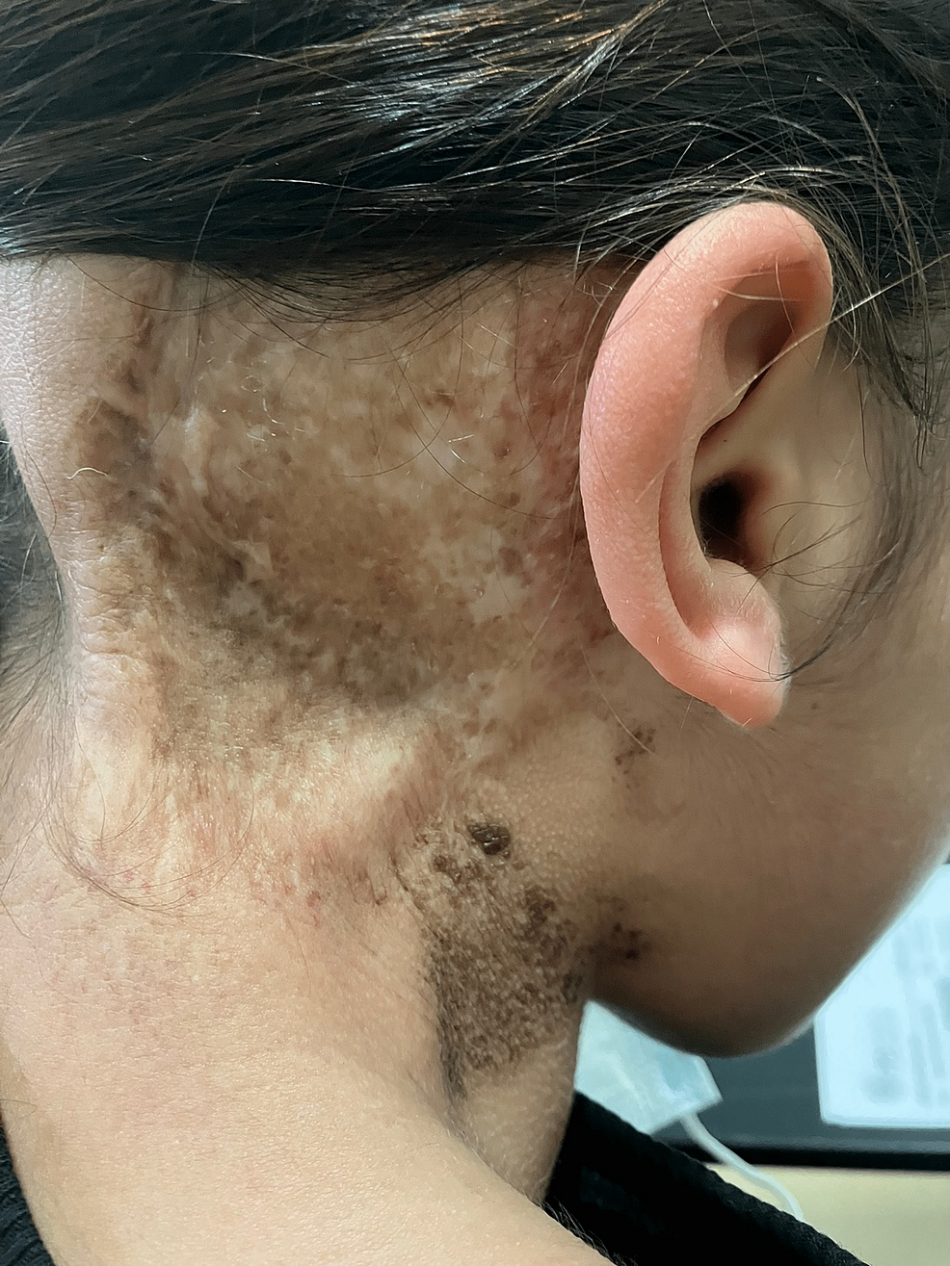
Retroauricular lesion in December 2022.

The patient is on a regular follow-up to monitor disease status and treatment-related adverse events (Figure 7). By the end of January 2023, she was alive with no evidence of disease.

## Discussion

HC is a rare malignant tumor of the sweat glands, with few published data or treatment guidelines [4]. Most of HC treatment orientations are based on retrospective studies, clinical cases, and hospital experience [8]. The initially benign appearance of these lesions makes the final diagnosis a challenging feat.

In this case, initially, the lesion was classified as a benign tumor, and then, due to multiple recurrences and fast growth, as an undifferentiated carcinoma with histological features of HC with areas of squamous, trichilemmal, and ductal differentiation. Some studies have reported that previously benign lesions, such as a hidradenoma, can evolve into malignant tumors [1,3]. We believe this is one of these cases after comparing the initial biopsy report with the later histopathological examination of the surgical specimen.

Surgical excision with wide margins (at least 2 cm, but, more recently, 3-5 cm are advised) and regional lymph node resection is the mainstay treatment [8,9]. Although, due to its location and anatomical boundaries, a wide-margin resection is not always possible. Therefore, there is a high probability of local recurrence.

External beam radiotherapy doses ranging from 42 to 66 Gy are typically used in the adjuvant setting. However, little data exist regarding the use of RT in metastatic HC. Despite remaining controversial, adjuvant radiotherapy to the surgical bed and regional lymph nodes may have an important role in local control, especially in high-risk circumstances such as deep invasion, positive margins, and extracapsular lymph node extension [10,11].

Although ChT is not currently used as an adjuvant treatment, it might be an option for irresectable or metastatic disease [12]. There is no true consensus on which regimen of ChT to use, but in selected cases, 5-fluorouracil, capecitabine, doxorubicin, platins, cyclophosphamide, vincristine, or bleomycin can be an option. Targeted therapies such as trastuzumab, epidermal growth factor receptor inhibitors, anti-programmed cell death-1 inhibitors, and PI3K/Akt/mTOR pathways are also under investigation [1,6,13].

## Conclusions

HC is a rare malignancy and difficult to diagnose. It must be discussed in a multidisciplinary group and addressed aggressively. Surgery may be the mainstay approach but RT and ChT, independently or in combination, must be considered in the presence of high-risk features. This pediatric patient was treated with wide local excision with ipsilateral neck dissection, followed by RT. Effective local control was achieved. However, more robust clinical evidence is needed to define the best treatment approach for these aggressive tumors.

## References

[1] Soni A, Bansal N, Kaushal V, Chauhan AK (2015). Current management approach to hidradenocarcinoma: a comprehensive review of the literature. Ecancermedicalscience.

[2] Gao T, Pan S, Li M, Su R (2022). Prognostic analysis of hidradenocarcinoma: a SEER-based observational study. Ann Med.

[3] Gouiaa N, Abbes K, Fakhfekh I (2008). [Hidradenocarcinoma arising from pre-existing hidradenoma]. Ann Dermatol Venereol.

[4] de Lima AA, Santos M, de Morais PM, Rodrigues CA (2021). Hidradenocarcinoma. An Bras Dermatol.

[5] Gupta E, Guthrie KJ, Krishna M, Asmann Y, Parker AS, Joseph RW (2015). Whole exome sequencing of a patient with metastatic hidradenocarcinoma and review of the literature. Rare Tumors.

[6] Guillot B (2009). Unusual cutaneous malignancies: cutaneous adnexal tumours. Management of Rare Adult Tumours.

[7] Harari PM, Shimm DS, Bangert JL, Cassady JR (1990). The role of radiotherapy in the treatment of malignant sweat gland neoplasms. Cancer.

[8] Hall J, Knee G, A'Hern RP, Clarke J, Glees JP, Ford HT, Eeles RA (2006). Sweat-gland tumours: a clinical review of cases in one centre over 20 years. Clin Oncol (R Coll Radiol).

[9] Martins D, Pereira F, Azevedo R, Julião I (2022). Eccrine hidradenocarcinoma of the scalp. Cureus.

[10] Mir Khan B, Mansha MA, Ali N, Abbasi AN, Ahmed SM, Qureshi BM (2018). Hidradenocarcinoma: five years of local and systemic control of a rare sweat gland neoplasm with nodal metastasis. Cureus.

[11] Miller DH, Peterson JL, Buskirk SJ (2015). Management of metastatic apocrine hidradenocarcinoma with chemotherapy and radiation. Rare Tumors.

[12] Lerner A, Beckford A, Ugent S, Goldberg L, Jalisi S, Demierre MF (2011). Complete response of metastatic malignant hidradenocarcinoma to capecitabine treatment. Arch Dermatol.

[13] Amel T, Olfa G, Faten H, Makrem H, Slim BA, Moncef M (2009). Metastatic hidradenocarcinoma: surgery and chemotherapy. N Am J Med Sci.

